# Incorporating radiomic feature of pretreatment ^18^F-FDG PET improves survival stratification in patients with EGFR-mutated lung adenocarcinoma

**DOI:** 10.1371/journal.pone.0244502

**Published:** 2020-12-28

**Authors:** Yu-Hung Chen, Tso-Fu Wang, Sung-Chao Chu, Chih-Bin Lin, Ling-Yi Wang, Kun-Han Lue, Shu-Hsin Liu, Sheng-Chieh Chan

**Affiliations:** 1 Department of Nuclear Medicine, Hualien Tzu Chi Hospital, Buddhist Tzu Chi Medical Foundation, Hualien, Taiwan; 2 Department of Medicine, College of Medicine, Tzu Chi University, Hualien, Taiwan; 3 Department of Hematology and Oncology, Hualien Tzu Chi Hospital, Buddhist Tzu Chi Medical Foundation, Hualien, Taiwan; 4 Department of Internal Medicine, Hualien Tzu Chi Hospital, Buddhist Tzu Chi Medical Foundation, Hualien, Taiwan; 5 Epidemiology and Biostatistics Consulting Center, Department of Medical Research and Department of Pharmacy, Tzu Chi General Hospital, Hualien, Taiwan; 6 Department of Medical Imaging and Radiological Sciences, Tzu Chi University of Science and Technology, Hualien, Taiwan; Spedali Civili of Brescia, University of Brescia, ITALY

## Abstract

**Background:**

To investigate the survival prognostic value of the radiomic features of ^18^F-FDG PET in patients who had EGFR (epidermal growth factor receptor) mutated lung adenocarcinoma and received targeted TKI (tyrosine kinase inhibitor) treatment.

**Methods:**

Fifty-one patients with stage III-IV lung adenocarcinoma and actionable EGFR mutation who received first-line TKI were retrospectively analyzed. All patients underwent pretreatment ^18^F-FDG PET/CT, and we calculated the PET-derived radiomic features. Cox proportional hazard model was used to examine the association between the radiomic features and the survival outcomes, including progression-free survival (PFS) and overall survival (OS). A score model was established according to the independent prognostic predictors and we compared this model to the TNM staging system using Harrell's concordance index (c-index).

**Results:**

Forty-eight patients (94.1%) experienced disease progression and 41 patients (80.4%) died. Primary tumor SUV entropy > 5.36, and presence of pleural effusion were independently associated with worse OS (both *p* < 0.001) and PFS (*p* = 0.001, and 0.003, respectively). We used these two survival predictors to devise a scoring system (score 0–2). Patients with a score of 1 or 2 had a worse survival than those with a score of 0 (HR for OS: 3.6, *p* = 0.006 for score 1, and HR: 21.8, *p* < 0.001 for score 2; HR for PFS: 2.2, *p* = 0.027 for score 1 and HR: 8.8, *p* < 0.001 for score 2). Our scoring system surpassed the TNM staging system (c-index = 0.691 versus 0.574, *p* = 0.013 for OS, and c-index = 0.649 versus 0.517, *p* = 0.004 for PFS).

**Conclusions:**

In this preliminary study, combining PET radiomics with clinical risk factors may improve survival stratification in stage III-IV lung adenocarcinoma with actionable EFGR mutation. Our proposed scoring system may assist with optimization of individualized treatment strategies in these patients.

## Introduction

The incidence of lung cancer is the highest among all types of cancers. Lung cancer is also the leading cause of cancer-related deaths worldwide [1]. In the United States, approximately 230000 new lung cancer cases, and nearly 143000 lung cancer related deaths were reported in the 2019 cancer statistics [1, 2]. Adenocarcinoma is currently the most common histopathological subtype of lung cancer, and the incidence is rising [3]. The majority of lung adenocarcinoma cases present as metastatic disease upon diagnosis, and the prognosis among these patients is grim [1–4]. Fortunately, several targetable driver mutations have been discovered, and the use of the corresponding targeted therapeutic agents has significantly improved the prognosis of metastatic lung adenocarcinoma [5–8]. Among these targetable driver mutations, epidermal growth factor receptor (EGFR) mutation is the most common, and many effective tyrosine kinase inhibitors (TKIs) have been introduced [5, 6]. Although novel TKIs for EGFR mutation have emerged, 20%–40% of patients are non-responsive to TKI. Even though approximately 58%–83% of the cases are responsive initially, 50%–65% of cases will acquire resistance within 1 year [6]. Currently, drug resistance in patients with EGFR mutation cannot be accurately anticipated; as such, a reliable prediction tool is an imperative and unmet need [9].

^18^F-fluorodeoxyglucose (^18^F-FDG) positron emission tomography (PET) is an imaging modality that is capable of spotting altered glycolytic activity. It has been used widely as a staging, re-staging, and response evaluation tool for lung adenocarcinoma [10, 11]. Literature has shown that several ^18^F-FDG PET derived semiquantitative parameters such as the standardized uptake value (SUV), metabolic tumor volume (MTV), and total lesion glycolysis (TLG), are associated with the prognosis in patients with non-small cell lung cancer [12–14]. In addition, texture analysis provides a new way of featuring for ^18^F-FDG PET, and some reports have shown an association between the texture features of ^18^F-FDG PET and the tumor control and survival of patients with lung cancer [15–17]. The texture features of ^18^F-FDG PET allow us to assess the heterogeneity of a tumor, which is driven by genomic diversity that allows the tumors to adapt and fight against treatments. Therefore, tumor heterogeneity is associated with the prognosis of cancers [18–21]. Texture features and semiquantitative parameters are radiomic features of ^18^F-FDG PET. Distinct from the current cancer staging system and clinical risk factors, which focus more on the description of the disease extent or invasiveness, radiomics portrays more about the tumor biology and heterogeneity [4, 22, 23]. In this context, radiomics may complement the prognostic strength of the current clinical risk factors.

We conducted this study to investigate the utility of radiomic features of ^18^F-FDG PET in predicting the survival in patients with primary advanced EGFR-mutated lung adenocarcinoma treated with TKIs.

## Materials and methods

### Study population

The Research Ethics Committee of Hualien Tzu Chi Hospital, Buddhist Tzu Chi Medical Foundation approved the protocol of this retrospective study (IRB109-010-B). The requirement of informed consent for this study was waived. We retrospectively enrolled patients with newly diagnosed EGFR mutated lung adenocarcinoma from January 2010 to December 2014. All participants had pathologically proven lung adenocarcinoma and had undergone serial examinations for staging at the initial diagnosis; these included contrast-enhanced CT of the chest to upper abdomen, ^18^F-FDG PET/CT, and/or gadolinium-enhanced MRI of the brain. The staging was designated according to the 7th edition of the American Joint Committee on Cancer (AJCC) staging manual [4]. Patients with an AJCC stage of III or IV and an active EGFR mutation in exon 18, 19, 20, or 21 were included. All patients received EGFR targeting TKIs (Gefitinib, Erlotinib, or Afatinib) as the first-line treatment. Finally, 51 patients were included and followed until August 2019. The mutational analysis of EGFR was performed from the formalin-fixed, paraffin-embedded tissues of histopathologically confirmed lung adenocarcinoma. Mutations in EGFR were analyzed using an EGFR RGO Kit (Qiagen, Hilden, Germany). The choice of TKI was based on the decision of the attending physician. The examination findings were discussed at a multidisciplinary cancer conference convened by our thoracic oncology research group.

### Imaging protocol for ^18^F-FDG PET

All participants in our study fasted for at least 4 hours before ^18^F-FDG injection (400 MBq). The ^18^F-FDG PET/CT scans were acquired with the use of a GE Discovery ST PET/CT unit (Discovery ST16; GE Healthcare, Milwaukee, WI, USA). The PET/CT system was equipped with a PET unit having 10080 bismuth germanate crystals in 24 rings and a 16-detector row transmission CT unit (912 detectors/row). First, we acquired a transmission CT scan. The voltage and the current of the tube were 120 kV and 120 mA, respectively. The pitch of the transmission CT was 1.75. Image sampling was conducted using the helical mode with a helical thickness of 3.75 mm. The image reconstruction matrices were 512 × 512. No iodinated contrast material was administered for all transmission CT images. We obtained the PET images between 40 and 60 min after intravenous administration of radiotracer from the vertex to mid-thigh. The scanning time was 3.0 min for each table position (15 cm per table position with a 3-cm overlap for every contiguous frame). We used the transmission CT to perform attenuation correction for the PET images. The image reconstruction filter was ordered-subset expectation maximization iterative reconstruction algorithm (2 iterations and 21 subsets; matrix size, 128 × 128).

### The analysis of PET images

We used the PMOD 4.0 software package (PMOD Technologies Ltd., Zurich, Switzerland) to display the ^18^F-FDG PET/CT images and to perform the semiquantitative analysis. For semiquantitative analysis, we first identified the primary tumor of lung cancer on the ^18^F-FDG PET/CT image. An experienced nuclear medicine physician then drew a volume-of-interest for the primary tumor. The SUV of ^18^F-FDG PET was calculated and normalized based on the body weight:

$$SUV=\frac{\left(\text{decay-corrected}\;\text{activity}\;[\text{kBq}]\;\text{per}\;\text{milliliter}\;\text{of}\;\text{tissue}\;\text{volume}\right)}{\left(\text{injected}\;\text{FDG}\;\text{activity}[\text{kBq}]/\text{body}\;\text{weight}\;\text{in}\;\text{g}\right)}$$

We used an SUV threshold value of 2.5 for contouring the primary tumor inside the boundary of the volume-of-interest [17, 24]. The PMOD 4.0 software automatically generated the contour margin around the primary tumor inside the boundary. The voxels indicating the SUV value of > 2.5 within the contour margin were used to define the MTV. The mean SUV within the contour margin was automatically generated. The TLG was then calculated based on the following formula:

TLG=meanSUV×MTV

We used the contour margin of the primary tumor MTV to perform radiomic analysis. We selected three matrices to extract radiomic features from ^18^F-FDG PET images in our study, included SUV histogram analysis, the gray-level co-occurrence matrix (GLCM), and the gray-level size-zone matrix (GLSZM) [25–27]. For the analysis of GLCM and GLSZM, the ^18^F-FDG radioactivity uptake within the contour margin was resampled into 64 different values (bin number of 64) [17, 26, 28]. Subsequently, the radiomic features were computed as described in the previous studies [25–27]. Desseroit et al. reported the test-retest variabilities for the image features calculated from the SUV histogram, the GLCM, and the GLSZM in patients with non-small cell lung cancer. They found that the SUV entropy, the sum entropy, and small area emphasis (SAE) showed the least test-retest variability among the SUV histogram, the GLCM, and the GLSZM, respectively [29]. Therefore, we choose to use these three radiomic features for analysis in our study. The SUV entropy was defined as the uncertainty measure of the intensity distribution within the contour margin. P(*i*) indicated the probability of distinct resampled values and Ng represented the total number of discrete intensity levels within the contour margin. The ε is an arbitrarily small positive number (≈ 2.2 × 10^−16^). The calculation of SUV entropy was as follows:

$$SUV\;entropy=-\sum_{i=1}^{Ng}P(i)log2(P(i)+\varepsilon )$$

From GLCM, we extracted sum entropy (gray-level co-occurrence matrix sum entropy). When GLCM(*i*, *j*) represents the probability that a voxel intensity *i* is neighbor to another voxel of intensity *j*, and represents to be the discrete intensity levels within the contour margin. Px+y(k) can be determined as follows:

$$Px+y(k)=\sum_{i=1}^{Ng}\sum_{j=1}^{Ng}GLCM(i,j)$$

The *k* was *i + j*, and *k* = 2, 3, …, 2Ng. The sum entropy was calculated as follows:

$$Sum\;entropy=-\sum_{k=2}^{2Ng}Px+y(k)log2(Px+y(k)+\varepsilon )$$

Among the GLSZM parameters, SAE was selected for analysis. The matrix indicated how frequently a voxel of resampled intensity *i* was a size of *j* voxels. N*z* represents the number of zones in the contour margin, GLSZM(*i*,*j*) represents the probability of the intensity *i* being a zone size of *j*, while Ng and Ns represent the discrete intensity levels and the number of discrete zone sizes in the contour margin, respectively. The SAE was calculated as follows:

$$SAE=\frac{1}{\text{N}z}\sum_{i=1}^{Ng}\sum_{j=1}^{Ns}\frac{GLSZM(i,j)}{j2}$$

**Fig 1:**
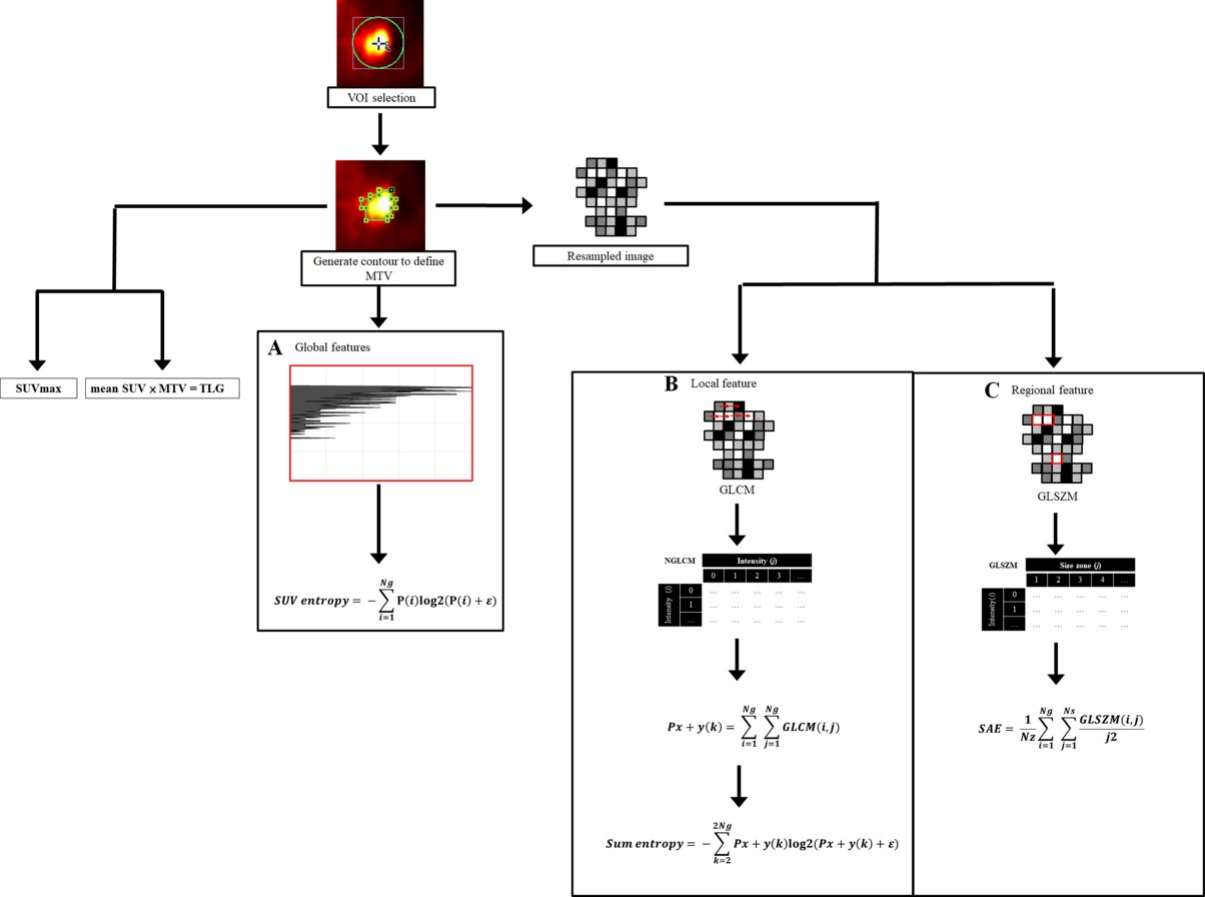
The method of radiomic analysis of ^18^F-FDG PET images.

We summarized the procedures of the radiomic analysis in Fig 1. The software used to execute the aforementioned radiomic features was Pyradiomics 2.2.0 [30].

We first placed a VOI to include the primary tumor, and then an SUV threshold value of 2.5 was used for contouring. SUV histogram and two matrices were used in our study: one for local feature, and one for regional feature. VOI: Volume-of-interest, SUV: Standardized uptake value, MTV: Metabolic tumor volume, GLCM: Gray-level co-occurrence matrix, GLSZM: Gray-level size zone matrix, P*i*: Probability of distinct resampled values, Ng: The total number of discrete intensity levels within contour margin, ε: An arbitrarily small positive number (≈ 2.2 × 10^−16^), GLCM(*i*,*j*): The probability that a voxel of intensity *i* is neighbor to another voxel of intensity *j*; *k* = *i* + *j*, and *k* = 2, 3, …, 2Ng, SAE: Small area emphasis, N*z*: Number of zones in the contour margin, Ns: Number of discrete zone sizes in the contour margin, GLSZM(*i*,*j*): The probability of the intensity *i* being a zone size of *j*.

### Follow-up of study patients

The results of the imaging study and the treatment strategies were discussed at a conference held by the thoracic oncology research group at our center. For lesions that were indicative of malignancy, image-guided biopsies were performed whenever possible. The patients were kept under close clinical and imaging follow-up if biopsy of a suspicious lesion was not feasible or if it yielded a negative result in a patient with equivocal or positive imaging findings. We followed-up the patient at the outpatient clinic at one-month interval, and we regularly performed thoracic-to-abdominal contrast-enhanced CT at 3-month intervals. When signs of symptoms of disease progression emerged, Contrast-enhanced CT, MRI of the brain and biopsy were performed. The treatment response to TKIs was classified as complete response, partial response, stable disease or progressive disease according to RECIST 1.1 criteria based on serial image studies [31]. New bloody effusion or a change from negative to positive fluid cytology was determined as disease progression.

### Data analysis

#### Analysis of survival outcome

All patients were followed-up until death or August 2019 (whichever occurred first). We expressed the demographic data as a frequency or mean and standard deviation, as appropriate. The overall survival (OS) was calculated from the date of diagnosis to the date of death or censored at the date of the last follow-up for surviving patients. Progression-free survival (PFS) was defined as the time between the start of TKI treatment and the date of disease progression (e.g., growth of a residual tumor or development of new metastatic lesion) or the date of death or censoring at the date of the last follow-up. The cut-off values for continuous variables were determined using the log-rank test based on the OS rates observed in the entire study cohort. We selected the cut-off values with the greatest chi-square value to be the optimal cut-off for each continuous variable [32]. An example of the operationalization of the cut-off value is shown in the S1 Fig. The optimal cut-off values for SUVmax, TLG, SUV entropy, sum entropy, and SAE were 6.75, 101, 5.36, 5.8, and 0.845, respectively. The association of the study variables with the survival outcomes was tested using the univariate and multivariate Cox regression analysis. We first examined the effect of each individual variable on the survival outcomes with the univariate analysis. Subsequently, the multivariate Cox regression analysis was used to identify the independent survival predictors. We expressed the results of the survival analysis as hazard ratios (HR) and 95% confidence intervals.

#### Model development

We established a scoring system to predict the OS and PFS based on the results of the multivariate Cox regression model. The presence of each independent survival predictor was designated a score of 1, and the absence of survival risk factor was assigned a score 0. For example, a patient with a score of 1 had one independent risk factor. We examined and compared the performance of our scoring system and the traditional AJCC staging system using the Kaplan-Meier curve method, log-rank test, and Harrell's concordance index (c-index) [33].

#### Model validation

The survival prediction model was validated using a bootstrapping validation method. The validation process was performed with 1000 bootstrap samples. The results of bootstrapping validation were expressed as bias with 95% confidence interval, standard error, and significance (*p*-value). Statistical analysis was performed using R-3.4.2 for Windows. The “compareC” package was applied to compare two correlated c-indices with a right-censored survival outcome. A two-tailed *p*-value of < 0.05 was considered statistically significant.

## Results

### Patient demographics

We summarized the distribution of patient characteristics of our study in the Table 1. A total of 39 (76.5%) patients were initially diagnosed with stage IV disease, 9 (17.6%) with stage IIIB disease, and 3 (5.9%) with stage IIIA disease. The three patients with stage IIIA status did not receive surgery as the primary treatment because of a poor pulmonary function test and/or advanced age. Twenty-six (51.0%) patients had L858R, 23 (45.0%) had a deletion in exon 19, and the other 2 patients had uncommon mutations; one with a S768I mutation and the other had a complex G719X mutation including G719A, G719S, G719C, and L861Q. The overall median follow-up period was 27.7 months (range 3.2–99.1 months) for all patients and 63.8 months (range 2.7.7–99.1 months) for the remaining 10 surviving patients. Forty-eight patients (94.1%) experienced disease progression during TKI treatment. A total of 41 (80.4%) patients had died by the time of the last follow-up. The 3-year OS rate and the 3-year PFS rate were 38.5% and 10.7%, respectively. The mean ± SD of the SUVmax and TLG values were 9.3 ± 3.65 and 216.5 ± 254.86, respectively. In terms of the radiomic features, the mean ± SD of the SUV entropy, sum entropy, and SAE were 5.3 ± 0.41, 5.8 ± 0.86 and 0.82 ± 0.590, respectively. A total of 44 (86.3%) patients experienced at least a partial response. The treatment response of first line TKI in 3 patients was stable disease, while the disease was progressive in 4 patients (Table 1).

**Table 1:** Baseline patient characteristics (n = 51).

Variable	Value
Age, years, mean ± SD	69 ± 9.0
Sex, n (%)	
Male	29 (59.6)
Female	22 (43.1)
Cigarette smoking, n (%)	
Ever smoker	21 (41.2)
Never smoker	30 (58.8)
Mutation type of EGFR	
Deletion 19	23 (45.0)
L858R	26 (51.0)
G719X	1 (2.0)
S768I	1 (2.0)
T classification, n (%)^a^	
T1	4 (7.8)
T2	16 (31.4)
T3	10 (19.6)
T4	21 (41.2)
N classification, n (%)^a^	
N0	7 (13.7)
N1	2 (4.0)
N2	17 (33.3)
N3	25 (49.0)
M classification, n (%)^a^	
M0	12 (23.5)
M1a	19 (37.3)
M1b	20 (39.2)
Overall stage, n (%)^a^	
Stage IIIA	3 (5.9)
Stage IIIB	9 (17.6)
Stage IV	39 (76.5)
Pleural effusion, n (%)	20 (39.2)
Lung to lung metastasis, n (%)	19 (37.3)
Hepatic metastasis, n (%)	6 (11.8)
Skeletal metastasis, n (%)	14 (27.5)
Brain metastasis, n (%)	6 (11.8)
First line TKI, n (%)	
Gefitinib	35 (68.6)
Erlotinib	12 (23.5)
Afatinib	4 (7.8)
Time from ^18^F-FDG PET to TKI treatment, day, median (IQR)	8 (24)
Response of treatment, n (%)^b^	
Complete remission	1 (2.0)
Partial response	43 (84.3)
Stable disease	3 (5.9)
Progressive disease	4 (7.8)

SD: Standard deviation, EGFR: Epidermal growth factor receptor, TKI: tyrosine kinase inhibitor, IQR: interquartile range.

^a^Staging was based on the 7^th^ American Joint Committee on Cancer system.

^b^Response assessment was based on RECIST 1.1 criteria.

### Univariate and multivariate Cox regression analysis

The median OS was 27.7 months (range, 3.2–99.1 months), and the median PFS was 14.1 months (range, 0–98.8 months). We summarized the results of the univariate and multivariate Cox regression analyses for the various study variables in the Table 2. The univariate analysis showed that poor OS was significantly associated with M1 status, presence of pleural effusion, presence of hepatic metastasis, a primary tumor SUVmax > 6.75, TLG > 101, a SUV entropy > 5.36, a sum entropy > 5.8, and a SAE ≤ 0.845. On the other hand, presence of pleural effusion and a SUV entropy > 5.36 were significantly associated with shorter PFS. The significant clinical and imaging variables in the univariate analysis were fitted into the multivariate Cox regression model. After multivariate analysis, the presence of pleural effusion, and a primary tumor SUV entropy > 5.36 were independent risk factors for both shorter OS and shorter PFS.

**Table 2:** Univariate and multivariate Cox regression analysis for prognostic factors of survival outcome (n = 51).

Variable	No.	OS				PFS			
		Univariate		Multivariate		Univariate		Multivariate	
		HR (95% CI)	*p*-value	HR (95% CI)	*p*-value	HR (95% CI)	*p*-value	HR (95% CI)	*p*-value
T4 disease			0.977		NA		0.726		NA
Yes	21	1.0 (0.5–1.9)				1.1 (0.6–2.0)			
No	30	Reference				Reference			
N3 disease			0.959		NA		0.091		NA
Yes	25	1.0 (0.5–1.9)				1.6 (0.9–2.9)			
No	26	Reference				Reference			
M1 disease			0.025		0.166		0.174		NA
Yes	39	2.7 (1.1–6.6)				1.6 (0.8–3.4)			
No	12	Reference				Reference			
Mutation type			0.261		NA		0.467		NA
Deletion 19	23	0.7 (0.4–1.3)				0.8 (0.4–1.4)			
Others	28	Reference				Reference			
Pleural effusion			0.037		< 0.001		0.044		0.003
Presence	20	2.0 (1.1–3.7)		4.0 (1.9–8.5)		1.8 (1.0–3.3)		2.7 (1.4–5.2)	
Absence	31	Reference		Reference		Reference		Reference	
Lung metastasis			0.938		NA		0.742		NA
Presence	19	1.0 (0.5–1.9)				1.1 (0.6–2.0)			
Absence	32	Reference				Reference			
Hepatic metastasis			0.026		0.903		0.234		NA
Presence	6	2.8 (1.1–6.9)				1.7 (0.7–4.2)			
Absence	45	Reference				Reference			
Skeletal metastasis			0.491		NA		0.267		NA
Presence	14	1.3 (0.6–2.6)				1.4 (0.8–2.7)			
Absence	37	Reference				Reference			
Brain metastasis			0.806		NA		0.579		NA
Presence	6	1.1 (0.4–3.2)				1.3 (0.5–3.0)			
Absence	45	Reference				Reference			
SUVmax			0.018		0.166		0.140		NA
> 6.75	13	2.7 (1.2–6.3)				1.7 (0.8–3.2)			
≤ 6.75	38	Reference				Reference			
TLG			0.002		0.739		0.074		NA
> 101	24	3.0 (1.5–5.9)				1.7 (1.0–3.0)			
≤ 101	27	Reference				Reference			
SUV entropy			0.002		<0.001		0.018		0.001
> 5.36	27	2.9 (1.5–5.7)		5.5 (2.5–12.2)		2.0 (1.1–3.6)		2.9 (1.5–5.5)	
≤ 5.36	24	Reference		Reference		Reference		Reference	
Sum entropy			0.001		0.841		0.063		0.586
> 5.8	21	3.5 (1.7–7.2)				1.7 (1.0–3.1)			
≤ 5.8	30	Reference				Reference			
SAE			0.021		0.783		0.195		NA
≤ 0.845	34	2.3 (1.1–4.5)				1.5 (0.8–2.8)			
> 0.845	17	Reference				Reference			

HR: Hazard ratio, CI: Confidence interval, OS: Overall survival, PFS: Progression-free survival, TLG: Total lesion glycolysis, SAE: Small zone emphasis.

**Fig 2:**
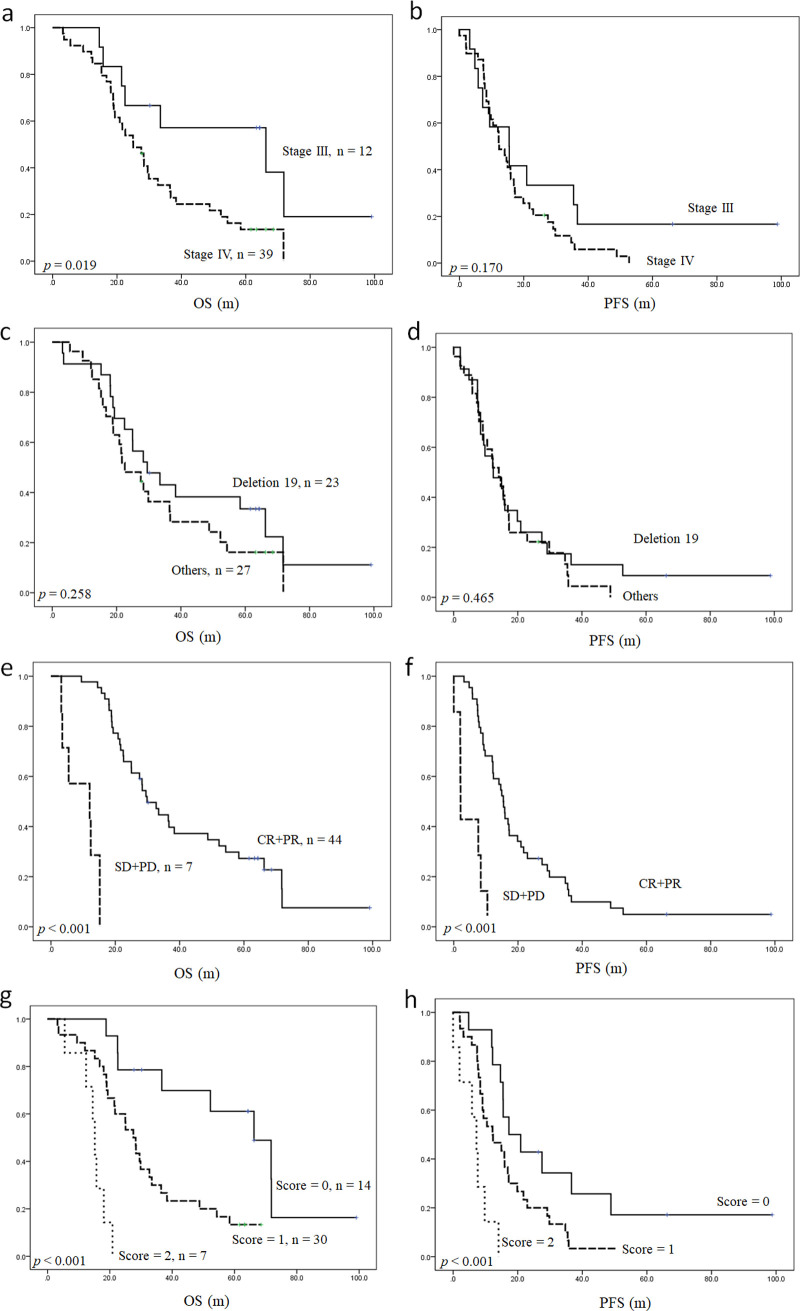
Kaplan-Meier curves presenting OS and PFS in patients with EGFR-mutated lung adenocarcinoma. Survival stratification according to the traditional TNM staging system (a and b), EGFR mutation status (c and d), treatment response based on RECIST 1.1 criteria (e and f), and the scoring system developed in the present study (g and h). OS: Overall survival, PFS: Progression-free survival, EGFR: Epidermal growth factor receptor, CR: Complete remission, PR: Partial response, SD: Stable disease, PD: Progressive disease.

**Fig 3:**
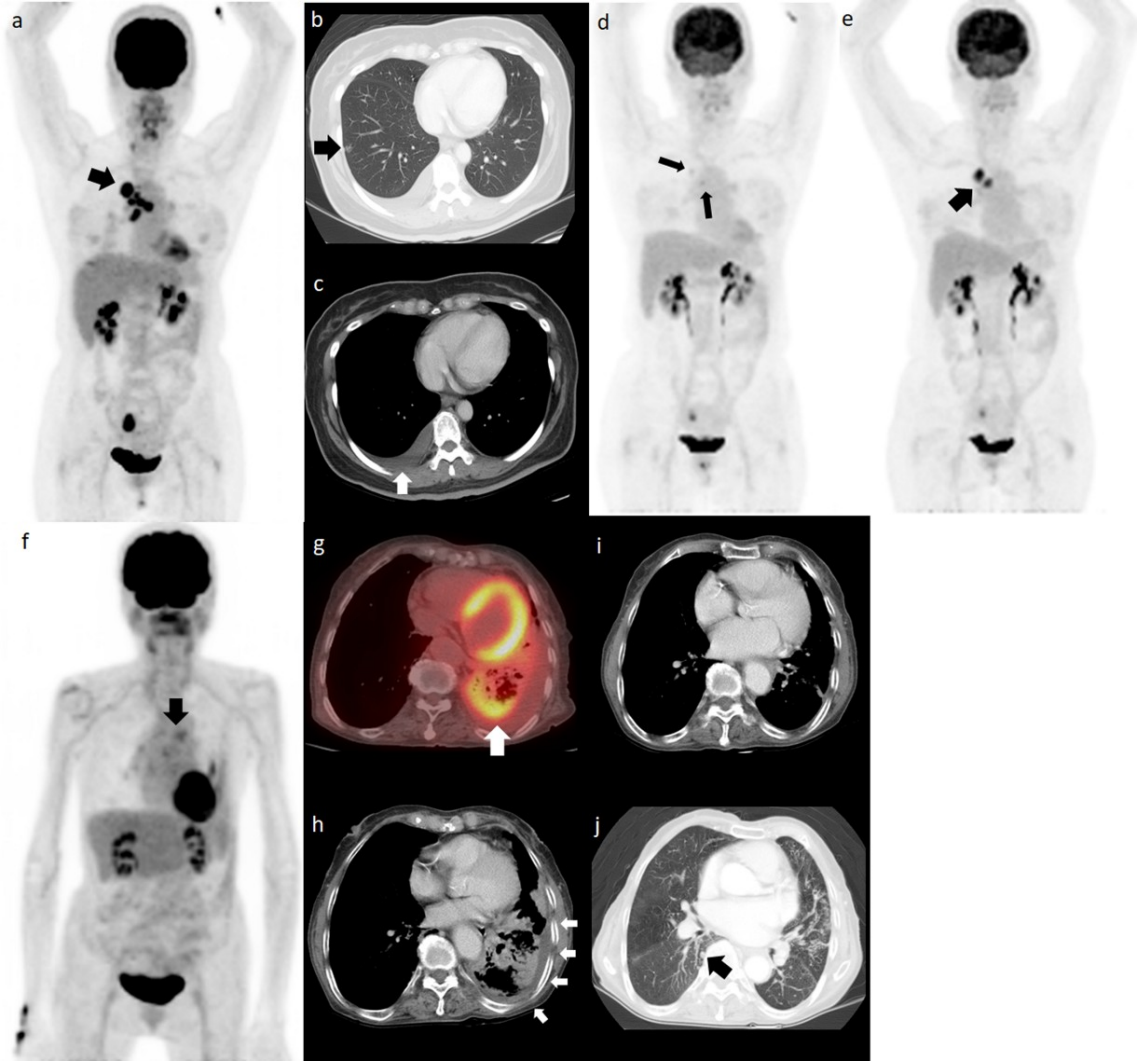
The survival prediction model outperforms the TNM staging system. Pretreatment ^18^F-FDG PET MIP image (a) for a 58-year-old woman with a primary tumor in the right upper lobe. Because of the presence of tiny nodule in different ipsilateral lobe and malignant pleural effusion (b and c, arrow), the clinical staging was cT4N3M1a. The EGFR mutation was L858R. The SUV entropy of the primary tumor was 5.32 (0.04 lower than the cut-off). A score of 1 was assigned for this patient. After 1 year of erlotinib treatment, the ^18^F-FDG PET MIP image (d) showed remarkable resolution of the primary tumor and metastatic lymph node ^18^F-FDG uptake (arrows). The patient experienced disease progression after 29.8 months of first line erlotinib treatment (e, arrow) and the treatment changed to osimertinib thereafter. The patient died of lung cancer with a PFS and OS of 29.8 and 54.3 months, respectively. The pretreatment ^18^F-FDG PET/CT and MIP images from an 83-year-old woman revealed mediastinal lymph node metastases (f, arrow) and an ^18^F-FDG avid tumor in the left lower lung (g, arrow). The patient also demonstrated malignant pleural effusion (h, arrows). Her clinical staging was cT3N3M1a and she had a deletion 19 EGFR mutation. The primary tumor SUV entropy was 5.44 (0.08 higher than the cut-off) and a score of 2 was given for this patient. She received erlotinib as a first line therapy. The CT acquired at 6 months after erlotinib treatment (i) showed resolution of the left pleural fluid; however, a new pulmonary nodule developed in the contralateral lung after 9.8 months of erlotinib treatment (j, arrow). The patient died of lung cancer progression with an OS of 18.1 months. MIP: Maximum intensity projection, PET: Positron emission tomography, EGFR: Epidermal growth factor receptor, PFS: Progression-free survival, OS: Overall survival.

We devised a scoring system for predicting OS and PFS based on the number of independent risk factors present (presence of pleural effusion and primary tumor SUV entropy > 5.36). The presence or absence of each independent prognosticator was designated a score of 1 or 0, respectively, resulting in scores ranging from 0 to 2. Fig 2 demonstrates the value of this scoring system for stratifying OS and PFS in patients with EGFR-mutated lung adenocarcinoma. Compared with patients with a score of 0, the OS rates were worse in patients with a score of 1 (HR: 3.6, *p* = 0.006) and much worse in those with a score of 2 (HR: 21.8, *p* < 0.001). In the survival analysis of PFS, the PFS rates were also worse in patients with a score of 1 (HR: 2.2, *p* = 0.027) and much worse in those with a score of 2 (HR: 8.8, *p* < 0.001). Our survival prediction model outperformed the conventional TNM staging system and the survival stratification according to EGFR mutation status (Fig 3 and Table 3). Our model showed higher, yet non-statistically significant c-indices than the RECIST response assessment.

**Table 3:** Comparison of the c-indices of different survival prediction models (n = 51).

Model	c-index for OS	*p* value^a^	c-index for PFS	*p* value^a^
Our prediction model	0.691	NA	0.649	NA
TNM staging system^b^	0.574	0.013	0.517	0.004
Mutation type of EGFR^c^	0.549	0.003	0.515	0.001
Treatment response^d^	0.625	0.184	0.598	0.210

OS: Overall survival, PFS: Progression-free survival, NA: Not applicable.

^a^Compared to our prediction model.

^b^Staging was based on the 7^th^ American Joint Committee on Cancer system.

^c^Stratified according to the presence or absence of the exon 19 deletion.

^d^Response assessment was based on RECIST 1.1 criteria.

### Bootstrapping validation

We used the bootstrap method to validate our survival prediction model. The S1 Table presents the results of the bootstrap validation. The β estimates of the two variables (presence of pleural effusion and primary tumor SUV entropy > 5.36) were still statistically significant in predicting both OS and PFS in the bootstrap results.

## Discussion

Approximately 50%–65% of patients with EGFR-mutated lung adenocarcinoma will acquire resistance within 1 year despite initial response to TKI therapy, and eventually die of this disease [6]; thus, a more accurate prediction tool is currently an imperative need. ^18^F-FDG PET has shown some value in EGFR-mutated lung adenocarcinoma, and several studies have shown that image features extracted from ^18^F-FDG PET are associated with EGFR mutation status [34–36]. In addition, ^18^F-FDG PET derived radiomic features have been shown to anticipate the treatment response to TKIs in EGFR-mutated lung cancer [37–41]. However, the prognostic value of ^18^F-FDG PET-based radiomic features for the survival of lung cancer patients treated by EGFR-targeting TKIs has not been well investigated. In this study, we found that the SUV entropy measured from the pretreatment ^18^F-FDG PET was independently associated with the time-to-progression of EGFR-targeted TKI treatment and the OS in patients with lung adenocarcinoma harboring EGFR mutations. The combination of the ^18^F-FDG PET radiomic feature and clinical factor allows better survival stratification.

Since tumor heterogeneity results from genomic inhomogeneity and evolution of clones, targetable EGFR mutation may co-exist with non-targetable mutations such as T790M [6, 18, 42–44]. Therefore, the biopsy specimen may be inadequate, and it may be impossible to thoroughly study the EGFR mutation characteristics of the entire tumor. Inadequacy of biopsy has been demonstrated in the work of Kuiper et al., wherein it was reported that the T790M status in the re-biopsied lung cancer specimens showed contradictory results in 37% of cases [45]. In addition, according to the recent molecular-pathological profiling studies for advanced EGFR mutation-positive non-small cell lung cancer at baseline, the coexisting multiple genetic, phenotypic, and functional mechanisms may contribute to disease progression and cause intrinsic TKI resistance [46]. The advantage of extracting radiomics from ^18^F-FDG PET is the ability to analyze the entire tumor's heterogeneity, which circumvents the potential prejudice of focal biopsy. The radiomic features of ^18^F-FDG PET have been shown to correlate with genetic heterogeneity [47] and may serve as a surrogate marker for intratumoral heterogeneity or mutation. Higher heterogeneity may be associated with a higher likelihood of hidden non-targetable EGFR mutations. Therefore, radiomic analysis of ^18^F-FDG PET may surreptitiously pick out those with hidden non-targetable EGFR mutations and enable the stratification of patients into different risk groups.

Han et al. conducted a systemic review to summarize the prognostic value of radiomics of ^18^F-FDG PET in lung cancer. In their study, although many radiomics parameters showed prognostic significance, these parameters were not replicated across different studies [17]. Although discrepant results in the studies may be partially attributed to different cancer histology and AJCC staging status, the radiomic feature per se may also contribute to this discrepancy. Since radiomic features of ^18^F-FDG PET demonstrate diverse repeatability and reliability, selecting features with high reproducibility and high robustness is essential to ensure reliable data output [29, 48, 49]. In our study, the radiomic features selected for analysis have been reported to show high repeatability among different matrices [29]. We found that SUV entropy (a first-order feature) was an independent predictor of survival; this feature has been reported to be repeatable in different image reconstruction methods and resampling methods, and showed good interclass correlation in the test-retest examination [50–52].

The presence of pleural effusion was another independent prognostic factor for OS and PFS in our study. The prognostic value of pleural effusion has been reported in both non-small cell lung cancer and small cell lung cancer [22, 23, 53, 54]. Pleural effusion in patients with lung cancer can directly result from pleural invasion or an indirect consequence of other pathological processes such as mediastinal nodal involvement. Effusion without tumor cell on the pathological exam may also represent an early phase in the development of malignant pleural effusion [23, 53, 55]. In addition, the work by Chen et al.’s showed that the cancer stem cells in lung adenocarcinoma-related pleural effusion were associated with distant metastasis and unfavorable survival. The negative prognostic effect of cancer stem cells might result from the epithelial-mesenchymal transition and adaptation in the cancer microenvironment [56]. Therefore, the presence of pleural effusion may be regarded as a clinical factor of tumor invasion, metastasis, and a more substantial disease burden.

The presence of pleural effusion (clinical factor) and the radiomics derived from the ^18^F-FDG PET represent distinct biological characteristics of cancer. Pleural effusion reflects tumor invasiveness in the microenvironment, as well as the disease burden [22, 23, 53–56]. In contrast, radiomic feature describes heterogeneity, and is closely related to tumor evolution [18, 19]. Based on these philosophies, the clinical factor and radiomic feature of ^18^F-FDG PET may be complementary in anticipating the survival prognosis. Therefore, incorporating the two factors may achieve a better survival prediction. However, the prognostic role of combining clinical factor and radiomics of ^18^F-FDG PET in EGFR-mutated lung cancer has not been thoroughly studied. We found that the combination of pleural effusion and pretreatment primary tumor SUV entropy improved survival stratification. Based on this, we developed a model with a score of 0–2 for predicting OS and PFS according to the number of independent prognosticators present. This survival model showed a significantly higher capability to stratify the survival of patients than the traditional AJCC staging system and the EGFR mutation status. The predictive power of our model was also better than the RECIST system (Table 3), which offers an opportunity to select a risk-directed treatment strategy earlier.

**Fig 4:**
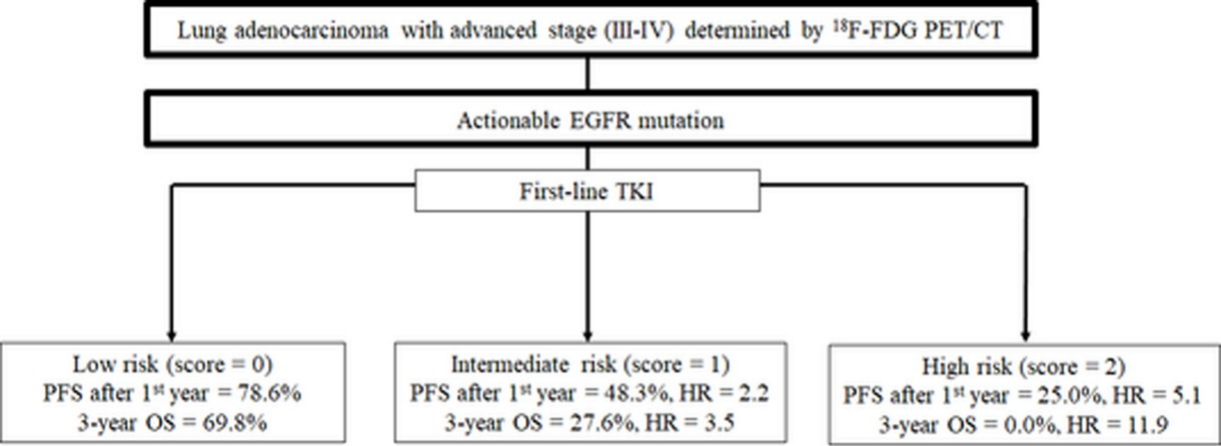
Flow chart illustrating the potential utility of our scoring system in the management of patients with EGFR-mutated lung adenocarcinoma treated with TKI. EGFR: Epidermal growth factor receptor, TKI: Tyrosine kinase inhibitor, PFS: Progression-free survival, OS: Overall survival, HR: Hazard ratio.

Currently, the time-to-progression of TKI therapy in actionable EGFR mutations is heterogeneous, and there is no reliable model to predict drug resistance or treatment failure before the start of TKI treatment [6]. New therapeutic strategies have been designed to overcome resistance and improve survival time. For example, initial treatment with the third-generation TKI, osimertinib has gained success in improving survival in EGFR-mutated lung cancer, possibly due to the suppression of tumors harboring hidden T790M mutation [57]. Moreover, the addition of anti-angiogenesis agents to standard TKI also shows PFS benefit [58–60]. In addition, a recent study discovered that the TKI resistance pathway is associated with increased PD-L1 (programmed death-ligand 1) expression [61]. Treatment combination with immune modulation may become another choice [62]. However, in the era of precision medicine, more sophisticated patient stratification is pivotal, and add-on therapy in patients with an excellent response to standard TKI may be obviated. Therefore, our prediction model may be used to stratify patients into different risk groups before the initiation of TKI therapy (Fig 4).

Cook et al. have reported that the interval change of ^18^F-FDG PET radiomic feature, instead of pretreatment feature, independently predicted the early treatment response to TKI at 12 weeks [41]. Our data are in agreement with these results and showed that pretreatment radiomic features were not significantly associated with the early response (S2 Table). Additional studies with a larger study population are required to investigate whether the combination of pretreatment and interim ^18^F-FDG PET may further refine the prognostic stratification for both treatment response and survival of lung cancer patients treated with TKIs.

There were several limitations in our study. First, our study cohort was relatively small. Second, this was a retrospective study, and we can not avoid the bias associated with the retrospective review process. Finally, we only performed internal validation for our survival prediction model. The generalizability of our survival prediction model should be prospectively validated using a larger external cohort.

## Conclusion

The preliminary data of our study indicate that primary tumor SUV entropy and pleural effusion were early predictive biomarkers of survival in patients with lung adenocarcinoma treated with EGFR-targeted TKIs. The combination of this PET radiomic feature with clinical risk factor yielded a better prognostic stratification model. Our proposed scoring model may enable tailored treatment approaches in patients with EGFR-mutated lung adenocarcinoma treated with standard TKI.

## Supporting information

S1 Fig An example of obtaining the cut-off value from a continuous variable. We tested different cut-off values for the SUV entropy using the log-rank test based on the overall survival rates. Serial chi-square values were obtained, and we choose the cut-off value with the highest chi-square value for further analysis (arrow, SUV entropy cut-off at 5.36 with a chi-square value of 9.82).
(TIF)

S1 Table Results of the bootstrapping validation of the prediction model. OS: Overall survival, PFS: Progression-free survival, 95% CI: Bias corrected accelerated 95% confidence interval, SE: Standard error.
(DOCX)

S2 Table Association of clinical and pretreatment imaging variables with the treatment response (n = 51). SD: Stable disease, PD: Progressive disease, OR: Odds ratio, NA: Not applicable. ^a^Response assessment was based on RECIST 1.1 criteria. ^b^Fisher’s exact test.
(DOCX)
