# Supplementary figures and images for: Incorporating radiomic feature of pretreatment 18F-FDG PET improves survival stratification in patients with EGFR-mutated lung adenocarcinoma
Source: PLoS One. 2020 Dec 28;15(12):e0244502. doi: 10.1371/journal.pone.0244502 (PMC7769431; doi:10.1371/journal.pone.0244502)

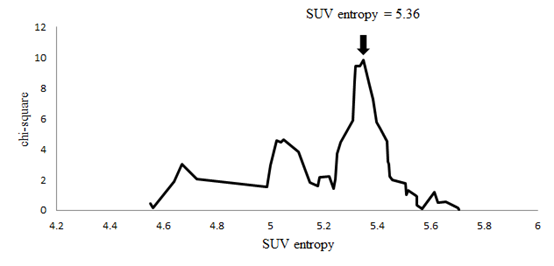

Supplement: S1 Fig — We tested different cut-off values for the SUV entropy using the log-rank test based on the overall survival rates. Serial chi-square values were obtained, and we choose the cut-off value with the highest chi-square value for further analysis (arrow, SUV entropy cut-off at 5.36 with a chi-square value of 9.82). (TIF) [file pone.0244502.s001.tif]
