# Supplementary material for: Incorporating radiomic feature of pretreatment 18F-FDG PET improves survival stratification in patients with EGFR-mutated lung adenocarcinoma
Source: PLoS One. 2020 Dec 28;15(12):e0244502. doi: 10.1371/journal.pone.0244502 (PMC7769431; doi:10.1371/journal.pone.0244502)
Supplement: S1 Table — OS: Overall survival, PFS: Progression-free survival, 95% CI: Bias corrected accelerated 95% confidence interval, SE: Standard error. (DOCX) [file pone.0244502.s002.docx]

**S1**

Incorporating radiomic feature of pre-treatment ^18^F-FDG PET improves survival stratification in patients with EGFR-mutated lung adenocarcinoma

Yu-Hung Chen, Tso-Fu Wang, Sung-Chao Chu, Chih-Bin Lin, Ling-Yi Wang, Kun-Han Lue, Shu-Hsin Liu, Sheng-Chieh Chan^*^

*Corresponding author

E-mail: williamsm.tw@gmail.com (SCC)

**S1 Table. Results of the bootstrapping validation of the prediction model**

|  | | β (95% CI) | Bias | SE | *p*-value |
| --- | --- | --- | --- | --- | --- |
| OS | Presence of pleural effusion | 1.4 (0.6–2.36) | 0.044 | 0.355 | 0.001 |
|  | SUV entropy > 5.36 | 1.7 (0.87–2.84) | 0.062 | 0.445 | 0.001 |
| PFS | Presence of pleural effusion | 1.0 (0.4–1.7) | 0.009 | 0.317 | 0.002 |
|  | SUV entropy > 5.36 | 1.1 (0.4–2.0) | 0.047 | 0.361 | 0.002 |

OS: Overall survival, PFS: Progression-free survival, 95% CI: Bias corrected accelerated 95% confidence interval, SE: Standard error.
