# Supplementary material for: Incorporating radiomic feature of pretreatment 18F-FDG PET improves survival stratification in patients with EGFR-mutated lung adenocarcinoma
Source: PLoS One. 2020 Dec 28;15(12):e0244502. doi: 10.1371/journal.pone.0244502 (PMC7769431; doi:10.1371/journal.pone.0244502)
Supplement: S2 Table — SD: Stable disease, PD: Progressive disease, OR: Odds ratio, NA: Not applicable. aResponse assessment was based on RECIST 1.1 criteria. bFisher’s exact test. (DOCX) [file pone.0244502.s003.docx]

**S2**

Incorporating radiomic feature of pre-treatment ^18^F-FDG PET improves survival stratification in patients with EGFR-mutated lung adenocarcinoma

Yu-Hung Chen, Tso-Fu Wang, Sung-Chao Chu, Chih-Bin Lin, Ling-Yi Wang, Kun-Han Lue, Shu-Hsin Liu, Sheng-Chieh Chan^*^

*Corresponding author

E-mail: williamsm.tw@gmail.com (SCC)

**S2 Table. Association of clinical and pretreatment imaging variables with treatment response (n = 51)**

| Variable | n | SD and PD^a^ | Univariate *p*-value^b^ | Multivariate (OR) | *p*-value |
| --- | --- | --- | --- | --- | --- |
| T classification |  |  | 0.427 |  | NA |
| 1–3 | 30 | 3 (10.0%) |  |  |  |
| 4 | 21 | 4 (19.0%) |  |  |  |
| N classification |  |  | 0.004* |  | 0.996 |
| 0–2 | 26 | 0 (0.0%) |  |  |  |
| 3 | 25 | 7 (28.0%) |  |  |  |
| M classification |  |  | 0.117 |  | NA |
| 0 | 12 | 0 (0.0%) |  |  |  |
| 1 | 39 | 7 (17.9%) |  |  |  |
| Pleural effusion |  |  | 0.096 |  | NA |
| Absence | 31 | 2 (6.5%) |  |  |  |
| Presence | 20 | 5 (25.0%) |  |  |  |
| Brain metastasis |  |  | 0.186 |  | NA |
| Absence | 45 | 5 (11.1%) |  |  |  |
| Presence | 6 | 2 (33.3%) |  |  |  |
| Liver metastasis |  |  | 0.028* |  | 0.997 |
| Absence | 45 | 4 (8.9%) |  |  |  |
| Presence | 6 | 3 (50.0%) |  |  |  |
| Skeletal metastasis |  |  | 0.08* |  | 0.765 |
| Absence | 37 | 3 (8.1%) |  |  |  |
| Presence | 14 | 4 (28.6%) |  |  |  |
| Lung-to-lung metastasis |  |  | 0.699 |  | NA |
| Absence | 19 | 2 (10.5%) |  |  |  |
| Presence | 32 | 5 (15.6%) |  |  |  |
| EGFR mutation |  |  | 1.000 |  | NA |
| Deletion 19 | 23 | 3 (13.0%) |  |  |  |
| Others | 27 | 4 (14.8%) |  |  |  |
| SUVmax |  |  | 0.662 |  | NA |
| < 6.75 | 13 | 1 (7.7%) |  |  |  |
| ≥ 6.75 | 38 | 6 (15.8%) |  |  |  |
| TLG |  |  | 0.103 |  | NA |
| < 101 | 24 | 1 (4.2%) |  |  |  |
| ≥ 101 | 27 | 6 (22.2%) |  |  |  |
| Histogram entropy |  |  | 0.232 |  | NA |
| < 5.36 | 27 | 2 (7.4%) |  |  |  |
| ≥ 5.36 | 24 | 5 (20.8%) |  |  |  |
| GLCM sum entropy |  |  | 0.033* |  | 0.998 |
| < 5.8 | 21 | 0 (0.0%) |  |  |  |
| ≥ 5.8 | 30 | 7 (23.3%) |  |  |  |
| SZE |  |  | 0.401 |  | NA |
| ≤ 0.845 | 34 | 6 (17.6%) |  |  |  |
| > 0.845 | 17 | 1 (5.9%) |  |  |  |

SD: Stable disease, PD: Progressive disease, OR: Odds ratio, NA: Not applicable.

^a^Response assessment was based on RECIST 1.1 criteria

^b^Fisher’s exact test
